# Treatment cost and life expectancy of diffuse large B-cell lymphoma (DLBCL): a discrete event simulation model on a UK population-based observational cohort

**DOI:** 10.1007/s10198-016-0775-4

**Published:** 2016-03-11

**Authors:** Han-I. Wang, Alexandra Smith, Eline Aas, Eve Roman, Simon Crouch, Cathy Burton, Russell Patmore

**Affiliations:** 1 Epidemiology and Cancer Statistics Group (ECSG), Department of Health Sciences, University of York, Seebohm Rowntree Building, Heslington, York, YO10 5DD UK; 2 Department of Health Management and Health Economics, University of Oslo, Oslo, Norway; 3 Haematological Malignancy Diagnostic Service, St. James’s University Hospital, Leeds, UK; 4 Queen’s Centre for Oncology and Haematology, Castle Hill Hospital, Hull, UK

**Keywords:** Diffuse large B-cell lymphoma, DLBCL, Cost, Discrete event simulation, Patient-level simulation, D24, D61, E17, H43, I11

## Abstract

**Background:**

Diffuse large B-cell lymphoma (DLBCL) is the commonest non-Hodgkin lymphoma. Previous studies examining the cost of treating DLBCL have generally focused on a specific first-line therapy alone; meaning that their findings can neither be extrapolated to the general patient population nor to other points along the treatment pathway. Based on empirical data from a representative population-based patient cohort, the objective of this study was to develop a simulation model that could predict costs and life expectancy of treating DLBCL.

**Methods:**

All patients newly diagnosed with DLBCL in the UK’s population-based Haematological Malignancy Research Network (www.hmrn.org) in 2007 were followed until 2013 (*n* = 271). Mapped treatment pathways, alongside cost information derived from the National Tariff 2013/14, were incorporated into a patient-level simulation model in order to reflect the heterogeneities of patient characteristics and treatment options. The NHS and social services perspective was adopted, and all outcomes were discounted at 3.5 % per annum.

**Results:**

Overall, the expected total medical costs were £22,122 for those treated with curative intent, and £2930 for those managed palliatively. For curative chemotherapy, the predicted medical costs were £14,966, £23,449 and £7376 for first-, second- and third-line treatments, respectively. The estimated annual cost for treating DLBCL across the UK was around £88–92 million.

**Conclusions:**

This is the first cost modelling study using empirical data to provide ‘real world’ evidence throughout the DLBCL treatment pathway. Future application of the model could include evaluation of new technologies/treatments to support healthcare decision makers, especially in the era of personalised medicine.

**Electronic supplementary material:**

The online version of this article (doi:10.1007/s10198-016-0775-4) contains supplementary material, which is available to authorized users.

## Introduction

With an annual incidence of around 10.2 per 100,000 in adults, diffuse large B-cell lymphoma (DLBCL) is the commonest lymphoma subtype, accounting for around 40 % of the total [1, 2]. Although rapidly fatal if left untreated, DLBCL is potentially curable [3]. Introduced in the 1970s, chemotherapy with cyclophosphamide combined with doxorubicin, vincristine and prednisone (CHOP) [4] resulted in a response rate of around 60 % and a long-term cure rate of 30 % [5, 6]; the addition of the monoclonal antibody rituximab in the 1990s increased the latter to 45 % [7].

Hitherto, although several economic studies have been carried out, the majority have focused on comparing the cost-effectiveness of CHOP and R-CHOP (CHOP plus rituximab) [8–17]. In addition, most of the relevant data has come from trials that only include patients treated with curative intent [9–12, 14, 16]; making findings difficult to extrapolate to the general patient population.

The objective of this study was to develop an economic model that could (1) model across the whole treatment pathway, rather than being limited to first-line treatment or a specific agent alone, (2) reflect real world practice rather than the idealized predefined setting of a randomised controlled trial, and (3) predict medical costs and life expectancy. Such a predictive disease model is particularly important for evaluating the cost-effectiveness of new interventions and for allocating health resources efficiently. To the best of our knowledge, no such model has been previously developed for DLBCL.

## Method

### Data sources

The individual-level data used for constructing the simulation model are from a specialist UK population-based registry, the Haematological Malignancy Research Network (www.hmrn.org); the methods of which have been previously described [1, 18]. Briefly, since September 2004, all patients newly diagnosed with a haematological malignancy (leukaemias, lymphomas, and myelomas) in a catchment population of more than 3.6 million have been routinely ascertained and followed-up. HMRN has Sect. 251 support under the NHS Act 2006, which allows all patients regardless of consent, to have full-treatment, response and outcome data collected to clinical trial standards; and to be ‘flagged’ for death and subsequent cancer registrations at the national Medical Research Information Service (MRIS) and linked to nationwide information on Hospital Episode Statistics (HES).

The current study includes all adult patients (≥18 years) newly diagnosed with de novo DLBCL (International Classification of Disease for Oncology, 3rd edition: 9680/3, 9735/3, 9712/3, and 9679/3) within HMRN in 2007 (*N* = 271). All patients were followed for 5 years from the date of diagnosis, and treatment pathways were individually mapped out according to the chemotherapy regimens received. A more detailed summary of patient characteristics is presented in Supplementary Table 1.

### Model structure

In order to reflect the current treatment strategies, while also being responsive to future changes, a discrete event based micro-simulation model was constructed using Simul8 software (Simul8 2013 Professional version, Simul8 Corporation, Boston, MA, USA). The model first assigned attributes (such as age at diagnosis, sex and prognostic factors) to a group of simulated patients, and then moved each patient forward to the next event, based both on their characteristics and on the timing of the events instead of fixed time cycles.

**Fig. 1:**
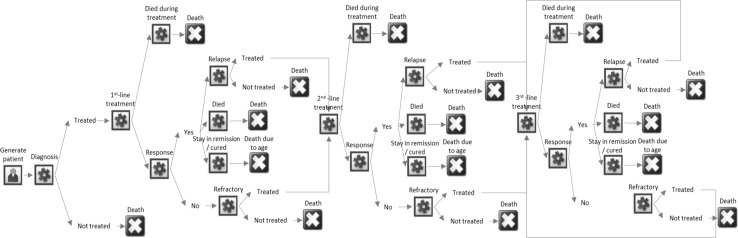
Model structure

The model structure was based on patient treatment pathways determined from empirical HMRN data, expert opinion and clinical guidelines. The structure of the model is shown in Fig. 1, and a simplified version of the model can be found via the following link https://www.hmrn.org/economics/models. Date of diagnosis defines the start of the model; with costs for diagnostic tests such as biopsies, scans, electrocardiography (ECG) and echocardiography (ECHO) being included. After diagnosis, the model splits into two branches according to whether the initial decision was to administer first-line chemotherapy with curative intent or manage supportively using a palliative approach. This is a unique and important feature of the model, ensuring the results reflect ‘real world’ practice and capture the fact that some patients are managed palliatively from the date of diagnosis until death. For those who entered the first-line curative treatment branch, different chemotherapy regimens, with or without supportive care, were assigned to each patient. The probability of receiving each treatment varied according to the patient’s individual attributes [such as age, disease stage and central nervous system (CNS) involvement]. This design allowed the model to capture the differences in ‘cost’ and ‘time in treatment’ between alternative regimens. However, it was beyond the scope of this study to compare the economic impact of different first-line chemotherapies.

Once first-line treatment had been received, one of three outcomes was assigned to each individual: died during treatment, responded to treatment, and no response to treatment. The probabilities of these outcomes were dependent on the first-line chemotherapy regimen and age at diagnosis.

For individuals who responded to first-line treatment, one of three events could occur: relapse, remain in remission until cured (defined as staying in remission ≥5 years) and death in remission. For those who were deemed to be cured, it was assumed that mortality returned to that of the general population and no subsequent DLBCL-related medical costs were incurred.

For individuals who relapsed or had disease that was refractory to treatment, two further options were possible: further potentially curative treatment or the adoption of an end-of-life (palliative) approach. The probability of the decision was dependent on age at diagnosis, previous chemotherapy regimen and response. For those who were not treated curatively, end-of-life care included all care given from last chemotherapy until death. For those who received second-line treatment, different types of chemotherapy regimens with or without autologous stem-cell transplant (ASCT) were included. Following this, each patient could remain in remission, receive third-line treatment, or receive end-of-life care; with the decision process being identical to that for first-line treatment. Few patients received treatment post third-line; so for the purposes of the model it was assumed that those who did had similar treatment patterns and response rates to those observed at third line.

The key input parameters used in the model are listed in Tables 1. For more details, please refer to Supplementary Tables 2–4.

**Table 1:** Key model parameters

Parameters	Estimates	Distribution	
Patient generation state			
Age (years)	Empirical	Beta	Mean (sd): 67.8 (14.7)
			Beta (3.73, 2.32)
Sex			
Age ≤40	Male: 0.56	Beta	*α* = 9, *β* = 7
Age 40–50	Male: 0.68	Beta	*α* = 15, *β* = 7
Age 50–60	Male: 0.71	Beta	*α* = 29, *β* = 12
Age 60–70	Male: 0.50	Beta	*α* = 31, *β* = 31
Age 70–80	Male: 0.55	Beta	*α* = 42, *β* = 35
Age ≥80	Male: 0.51	Beta	*α* = 27, *β* = 26
*Diagnosis*			
Diagnostic test results			
Age ≤40	Stage IA: 0.06	Dirichlet	*α*_1_ = 2
	CNS involvement: 0.0		*α*_2_ = 1
	Standard: 0.94		*α*_3_ = 16
	Died before diagnosis: 0.0		*α*_4_ = 2
Age 40–50	Stage IA: 0.09	Dirichlet	*α*_1_ = 3
	CNS involvement: 0.09		*α*_2_ = 3
	Standard: 0.77		*α*_3_ = 18
	Died before diagnosis: 0.05		*α*_4_ = 2
Age 50–60	Stage IA: 0.15	Dirichlet	*α*_1_ = 7
	CNS involvement: 0.08		*α*_2_ = 4
	Standard: 0.75		*α*_3_ = 32
	Died before diagnosis: 0.02		*α*_4_ = 2
Age 60–70	Stage IA: 0.06	Dirichlet	*α*_1_ = 5
	CNS involvement: 0.02		*α*_2_ = 2
	Standard: 0.90		*α*_3_ = 57
	Died before diagnosis: 0.02		*α*_4_ = 2
Age 70–80	Stage IA: 0.12	Dirichlet	*α*_1_ = 10
	CNS involvement: 0.01		*α*_2_ = 2
	Standard: 0.75		*α*_3_ = 59
	Died before diagnosis: 0.12		*α*_4_ = 10
Age ≥80	Stage IA: 0.05		*α*_1_ = 4
	CNS involvement: 0.00		*α*_2_ = 1
	Standard: 0.89		*α*_3_ = 48
	Died before diagnosis: 0.06		*α*_4_ = 4
Initial treatment decision			
Age ≤40	Treated: 0.94	Beta	*α* = 15, *β* = 1
Age 40–50	Treated: 0.95	Beta	*α* = 21, *β* = 1
Age 50–60	Treated: 0.96	Beta	*α* = 39, *β* = 2
Age 60–70	Treated: 0.88	Beta	*α* = 55, *β* = 7
Age 70–80	Treated: 0.75	Beta	*α* = 58, *β* = 19
Age ≥80	Treated: 0.54	Beta	*α* = 29, *β* = 24
Treatment decision for patients refractory to first-line chemotherapy			
Age ≤60	Treated: 0.86	Beta	*α* = 6, *β* = 1
Age 60–80	Treated: 0.33	Beta	*α* = 4, *β* = 8
Age ≥80	Treated: 0.00	Beta	*α* = 0, *β* = 6
Treatment decision for patients relapsed following first-line chemotherapy			
Age ≤60	Treated: 0.93	Beta	*α* = 14, *β* = 1
Age 60–80	Treated: 0.53	Beta	*α* = 8, *β* = 7
Age ≥80	Treated: 1.00	Beta	*α* = 2, *β* = 0
Treatment decision for patients refractory to second-line chemotherapy			
Age ≤60	Treated: 0.50	Beta	*α* = 4, *β* = 4
Age 60–80	Treated: 0.50	Beta	*α* = 3, *β* = 3
Age ≥80	Treated: 0.00	Beta	*α* = 0, *β* = 1
Treatment decision for patients relapsed following second-line chemotherapy			
Age ≤60	Treated: 0.50	Beta	*α* = 1, *β* = 1
Age 60–80	Treated: 0.00	Beta	*α* = 0, *β* = 1
Age ≥80	Treated: 0.00	Beta	*α* = 0, *β* = 1
Treatment decision for patients refractory to third-line chemotherapy			
Age ≤60	Treated: 0.50	Beta	*α* = 0, *β* = 3
Age 60–80	Treated: 0.00	Beta	*α* = 0, *β* = 1
Age ≥80	Treated: 0.00^a^	Beta	*α* = 0, *β* = 1
Treatment decision for patients relapsed following third-line chemotherapy			
Age ≤60	Treated: 0.50^a^	Beta	*α* = 1, *β* = 1
Age 60–80	Treated: 0.00^a^	Beta	*α* = 0, *β* = 1
Age ≥80	Treated: 0.00^a^	Beta	*α* = 0, *β* = 1

*CNS* central nervous system

^a^The probability was assumed to be the same as the probability in the second line due to lack of data

### Model inputs: medical costs

The model was built from an NHS perspective; and only medical costs directly related to DLBCL management were considered. This included costs for diagnosis, treatment, supportive care, follow-up and end-of-life care. Details of the cost items and different chemotherapy regimens included in each costing phase can be found in Supplementary Table 5.

All cost parameters were calculated using the National Tariff 2013/14 [19], representing the reimbursement/expenditure of NHS for treating the DLBCL population. For costs that were locally negotiated, such as the costs of chemotherapy regimens, information was derived from the Leeds Teaching Hospital NHS Trust. The inflated NHS reference cost 2012/13 [20] was used only when data were not available. All costs were expressed in 2013 British pound sterling; and the detailed cost information (unit costs) used in the model is summarised in Table 2.

**Table 2:** Summary of key unit costs

	Unit cost	Source
Inpatient stay		
Spell cost	£797	National Tariff 2013/14
Cost per excess bed day	£243	National Tariff 2013/14
Outpatient visit		
First attendance (single profession)	£247	National Tariff 2013/14
First attendance (multi-profession)	£248	National Tariff 2013/14
Follow-up visit (single profession)	£113	National Tariff 2013/14
Follow-up visit (multi-profession)	£174	National Tariff 2013/14
Diagnostic procedures		
Diagnostic biopsy	£260	National Tariff 2013/14^a^
Staging biopsy^b^	£503	National Tariff 2013/14
Imaging		
Computed tomography (CT)	£105	National Tariff 2013/14
Magnetic resonance imaging (MRI)	£206	National Tariff 2013/14
Positron emission tomography (PET)	£748	National Tariff 2013/14
Ultrasound	£51	National Tariff 2013/14
Electrocardiography (ECG)	£172	National Tariff 2013/14
Echocardiography (Echo)	£322	National Tariff 2013/14
Radiotherapy		
Planning	£769	National Tariff 2013/14
Per fraction	£123	National Tariff 2013/14
Chemotherapy (per cycle)		
CHOP	£289	Leeds Teaching Hospitals Trust
R-CHOP	£1730	Leeds Teaching Hospitals Trust
R-CVP	£1486	Leeds Teaching Hospitals Trust
CODOX-M/IVAC-R	£6241	Leeds Teaching Hospitals Trust
IDARAM	£2006	Leeds Teaching Hospitals Trust
R-DHAP	£1952	Leeds Teaching Hospitals Trust
R-ESHAP	£3344	Leeds Teaching Hospitals Trust
MiniBEAM	£446	Leeds Teaching Hospitals Trust
Methotrexate (low dose intrathecal)	£5	Leeds Teaching Hospitals Trust
Methotrexate (high dose)	£861	Leeds Teaching Hospitals Trust
Autologous stem-cell transplant (ASCT)	£42,000	Leeds Teaching Hospitals Trust

*CHOP* cyclophosphamide, doxorubicin, vincristine and prednisone, *R-CHOP* cyclophosphamide, doxorubicin, vincristine, prednisone and rituximab, *R-CVP* cyclophosphamide, vincristine, prednisone and rituximab, *CODOX-M/IVAC-R* cyclophosphamide, doxorubicin, vincristine, methotrexate/ifosfamide, etoposide, high dose cytarabine and rituximab, *IDARAM* rituximab, idarubicin, dexamethasone, cytarabine and methotrexate, *R-DHAP* dexamethasone, cytarabine, cisplatin and rituximab, *R-ESHAP* etoposide, methylprednisolone, cytarabine, cisplatin and rituximab, *MiniBEAM* carmustine, etoposide, cytarabine and melphalan

^a^Average of National Tariffs 2013/14

^b^Including: *BMAT* bone marrow aspirate and trephine, *BMA* bone marrow aspirate and *TB* bone marrow trephine

### Model inputs: time to event

**Fig. 2:**
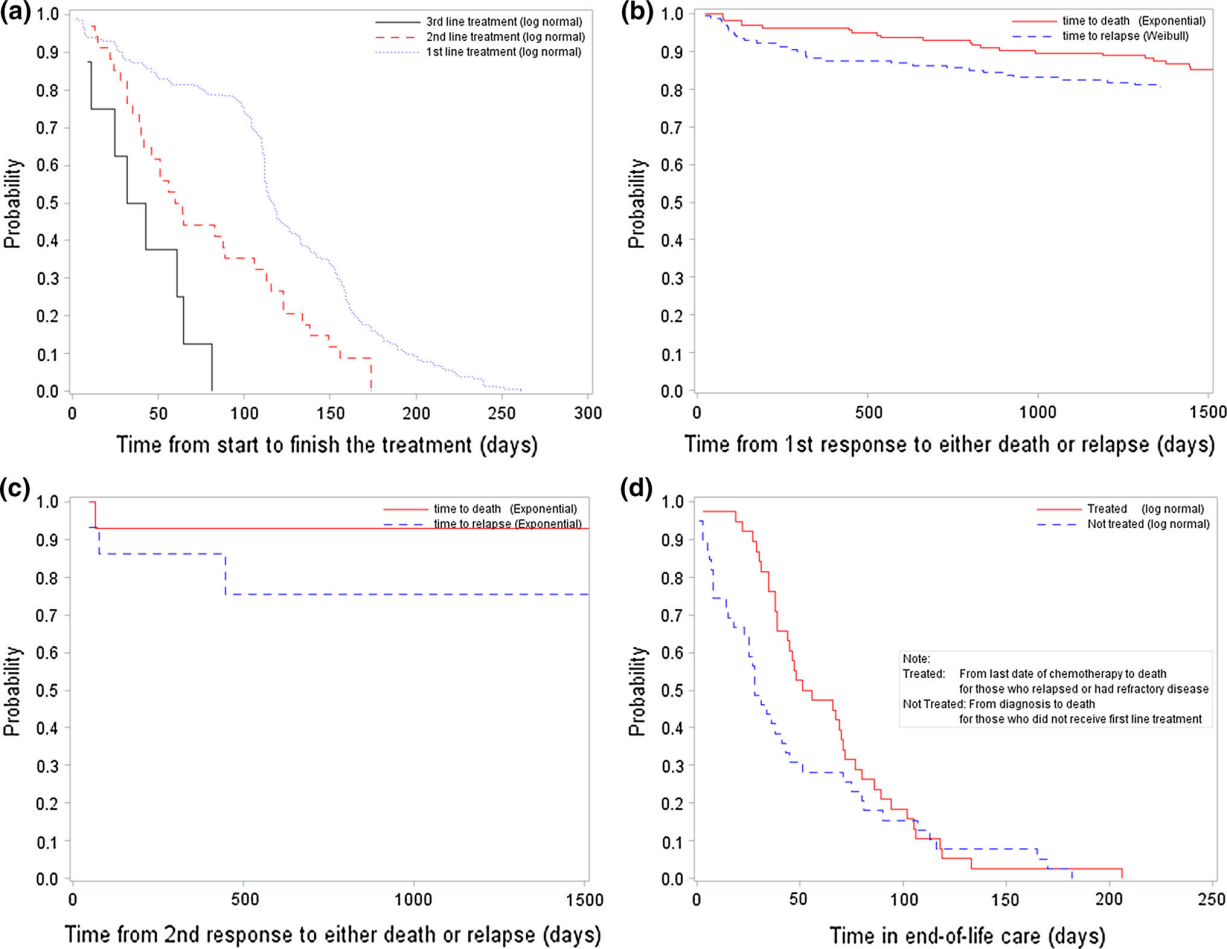
Time to event analyses. **a** Time in treatment. **b** Time in first response to either death or relapse. **c** Time in second response to either death or relapse. **d** Time in end-of-life care

Time-to-event (TTE) is a key element for discrete event simulation. Several time-to-event analyses were carried out using empirical data derived from HMRN to estimate the distributions associated with time between two events. This included the time from diagnosis to treatment, time in treatment, time from response to relapse, time from response to death and time in end-of-life care. All time-to-event analyses (survival analyses) were based on the best fit distributions as a function of patient’s age, treatment intent and treatment details. Five parametric survival models (exponential, Weibull, log-normal, log-logistic and Gompertz distributions) were tested and the best fit model was determined using the Akaike information criterion (AIC) score. It was assumed that cured patients’ mortality would return to normal, and the distribution of time to death was generated using the United Kingdom National Life Table, 2011–2013 [21]. The key parameters used in the model are illustrated in Fig. 2a–d. For more details on the time-to-event analyses, please refer to Supplementary Table 6.

### Model outputs

Health outcome was measured by life-years gained (LYG), while economic outcomes were captured by medical costs. Both economic and health outcomes were discounted using a 3.5 % annual discount rate, based on UK guidance recommended by the National Institute for Health and Clinical Excellence (NICE) [22].

### Assessing uncertainty

Probabilistic sensitivity analysis was performed on all parameters in order to explore the cumulative uncertainty of the model. Each parameter was assigned a distribution to reflect sample variability, whilst coefficients of survival models were assigned multivariate normal distributions. Then, Monte Carlo simulations were carried out by sampling parameters from the corresponding distributions simultaneously over a large number of iterations until stable results were reached (500 times). All outputs from the iterations were summarised with 95 % confidence intervals [23].

### Analysis

To investigate the impact on the UK as a whole, the annual number of expected cases in the UK (*N* = 4880) derived from HMRN rates was used to run the simulation model www.hmrn.org/statistics/incidence. Incidence-based results were presented in aggregate, as well as for the time horizons of 5-year, 15-year, and lifetime (simulated until 100 years of age or death). Survival beyond 5 years was extrapolated based on the best fit time-to-event distributions derived from the empirical data and the UK National Life Table, 2011–2013 [21]. See “Model inputs: time to event” in Methods for details.

To further investigate the effect of age, a sub-simulation was conducted to capture differences in cost and life-years gained for two age groups: under 70 and over 70 years of age. In addition, using the expected number of new cases of DLBCL diagnosed each year in the UK (*N* = 4880), the model further simulated national prevalence-based costs and life-years gained. Results were collected after a burn-in period of 10 years.

### Validation

The model was validated by means of standard methods, including face, internal and external validations [24]. Face validation was conducted while the model was under construction by consulting clinical experts on model structure, data sources and results. Internal validation was assessed by comparing predicted costs and life-years gained with empirical estimates, and external validation was carried out by comparing simulated results with relevant literature.

## Results

### Incidence-based results

Predicted costs, as well as life-days gained over 5-year, 15-year, and lifetime horizons are presented in Table 3 according to the simulation results of the model. Results for the 5-year horizon provide meaningful clinical estimates and allow internal validation, and results for the lifetime horizon provide insight into the overall economic and health impacts throughout the treatment pathway. Results over a 15-year time horizon are presented for the purpose of external comparison, as most published estimates are for this time period.

**Table 3:** Simulated medical costs and life-day gained of overall treatment pathway for DLBCL (*n* = 4880)

	*N*	5-year time horizon		*N*	15-year time horizon		*N*	Lifetime horizon	
		Cost (£), mean (95 % CI)	Life-days, mean (95 % CI)		Cost (£), mean (95 % CI)	Life-days, mean (95 % CI)		Cost (£), mean (95 % CI)	Life-days, mean (95 % CI)
Total	4880	18,096 (18,078–18,114)	1021 (1019–1022)	4880	18,396 (18,377–18,415)	2307 (2304–2310)	4880	18,396 (18,377–18,415)	3667 (3661–3672)
Treated	3892	21,712 (21,692–21,732)	1272 (1271–1273)	3892	22,122 (22,101–22,142)	2884 (2881–2887)	3892	22,122 (22,101–22,142)	4589 (4582–4596)
First-line only	3346	17,994 (17,985–18,003)	1300 (1299–1301)	3316	18,088 (18,078–18,097)	2990 (2987–2994)	3316	18,088 (18,078–18,097)	4718 (4711–4725)
Second-line plus									
With ASCT	154	78,273 (78,224–78,322)	1620 (1617–1622)	167	79,131 (79,084–79,178)	3705 (3692–3719)	167	79,131 (79,084–79,178)	6837 (6797–6877)
Without ASCT	392	31,209 (31,168–31,251)	889 (886–892)	409	31,471 (31,430–31,512)	1688 (1680–1696)	409	31,471 (31,430–31,512)	2628 (2610–2646)
Not treated	988	2930 (2918–2942)	30 (29–31)	988	2930 (2918–2942)	30 (29–31)	988	2930 (2918–2942)	30 (29–31)

*ASCT* autologous stem-cell transplant

Overall, the average cost per patient was around £18,000. This figure is consistent regardless of the time horizon chosen (£18,096, £18,396 and £18,396 for 5-year, 15-year and lifetime, respectively). This reflects the fact that for most DLBCL patients, treatment is completed within the 5-year time frame. However, the predicted life-years gained varied with time horizon: being 2.8, 6.3 and 10.0 for 5-year, 15-year and lifetime, respectively.

As expected, the costs for patients who received treatment with curative intent were significantly higher (£22,122), with more life-years gained (12.6 LYG) than for patients who were not treated (£2930, 0.1 LYG). This also applied to patients who received treatment post second-line. For patients who received ASCT as second-line treatment, costs were higher, but longer survival was observed (19.9 LYG and 7.6 LYG for ASCT and non-ASCT at second-line, respectively).

Table 4 shows the cost and time-to-next-event components of the overall treatment pathway over the lifetime horizon. As shown, treatment cost is the main component of the total costs. This is particularly prominent for second-line treatment involving ASCT (£56,442). For end-of-life care, patients who were not treated incurred less costs (£2930) than those who were treated prior to receiving end-of-life care (£4767).

**Table 4:** Costs and time-to-event breakdowns for treated DLBCL patients (based on life-time horizon)

	*N*	Cost (£)	Duration of event (days)
		Mean (95 % CI)	Mean (95 % CI)
Diagnosis	4880	1326 (1325–1327)	–
Treatment			
First-line treatment	3892	14,966 (14,958–15,974)	122 (121–123)
Second-line treatment	577	23,449 (23,365–23,534)	81 (80–82)
With ASCT	167	56,442 (56,409–56,474)	104 (103–105)
Without ASCT	409	9956 (9932–9981)	72 (71–73)
Third-line treatment	106	7376 (7374–7406)	50 (49–51)
Follow-up			
During first response	3001	1401 (1400–1402)	5125 (5117–5132)
During second response	296	1371 (1369–1374)	6135 (6106–6163)
End-of-life care			
For not treated patients	988	2930 (2918–2942)	30 (29–31)
For treated patients	704	4767 (4755–4780)	60 (60–61)

*ASCT* autologous stem-cell transplant

### Sub-group analysis

**Fig. 3:**
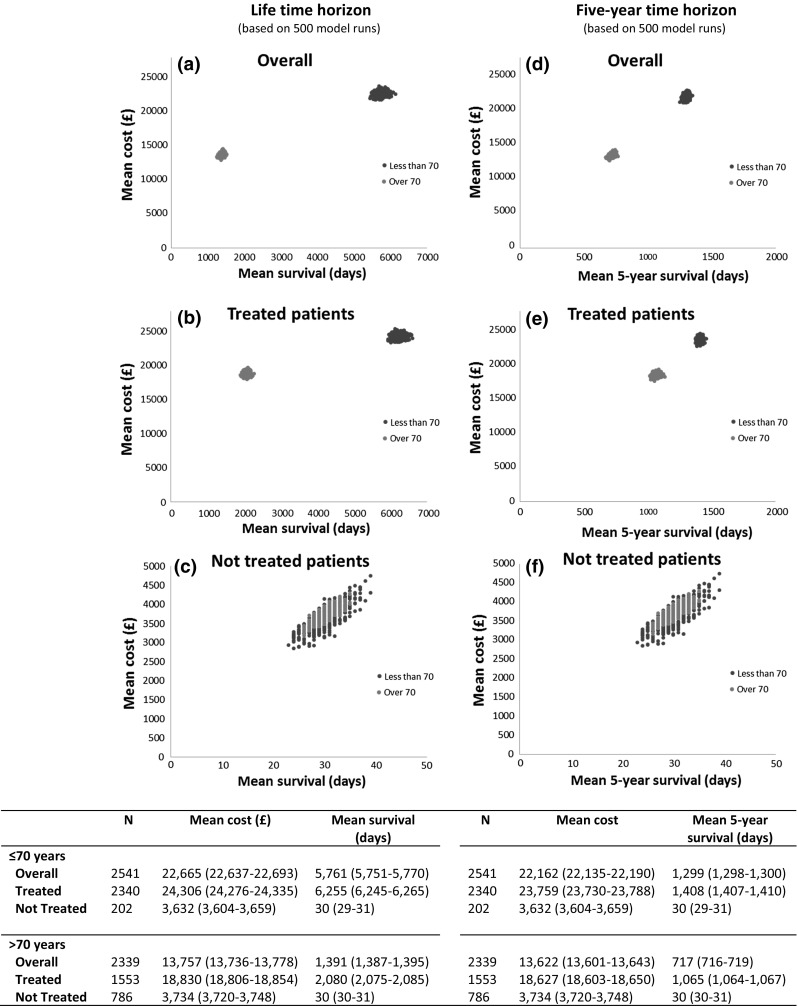
Simulated cost and survival between two age groups for three groups: **a** Overall. **b** Patients who received initial treatments and **c** patients who did not receive initial treatment over lifetime horizon, and **d** overall. **e** Patients who received initial treatments and **f** patients who did not receive initial treatment over 5-year time horizon

Figure 3 shows the effect of age for subgroups with different initial treatment intents. As expected, patients younger than 70 years had better survival but incurred more medical costs than those aged 70 years or more. However, for those who did not receive treatment with curative intent, medical costs and survival were similar between the two age groups.

Figure 3 also demonstrates the effect of time-horizon choice. As shown in Fig. 3b and e, among treated patients, survival differed between the two age groups (6255 and 2080 days over the lifetime). However, over a 5-year time horizon, the differences in life-years gained and costs were much smaller (1408 vs 1065 days); confirming that patients who were over the age of 70 responded as well as those who were younger.

### Prevalence-based results

**Fig. 4:**
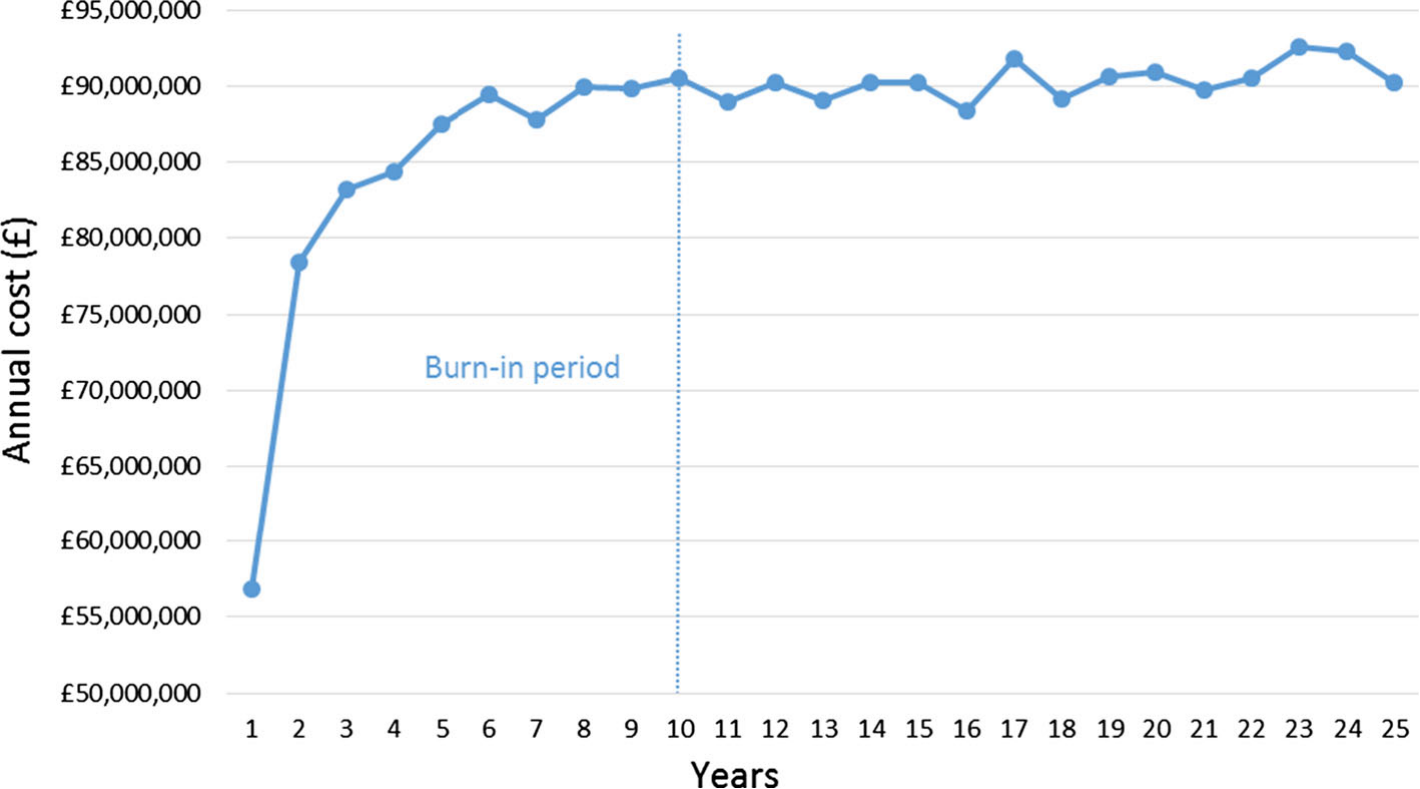
Annual cost (prevalence-based cost) for treating DLBCL population across the UK as a whole

The prevalence-based cost demonstrates the total cost associated with treating existing and new DLBCL patients during a 1-year period. The results are summarised in Fig. 4. As shown, the total annual costs for treating the DLBCL patient population across the UK as a whole was in the region of £88 million for the lower bound and £92 million for the upper bound.

### Model validation

**Fig. 5:**
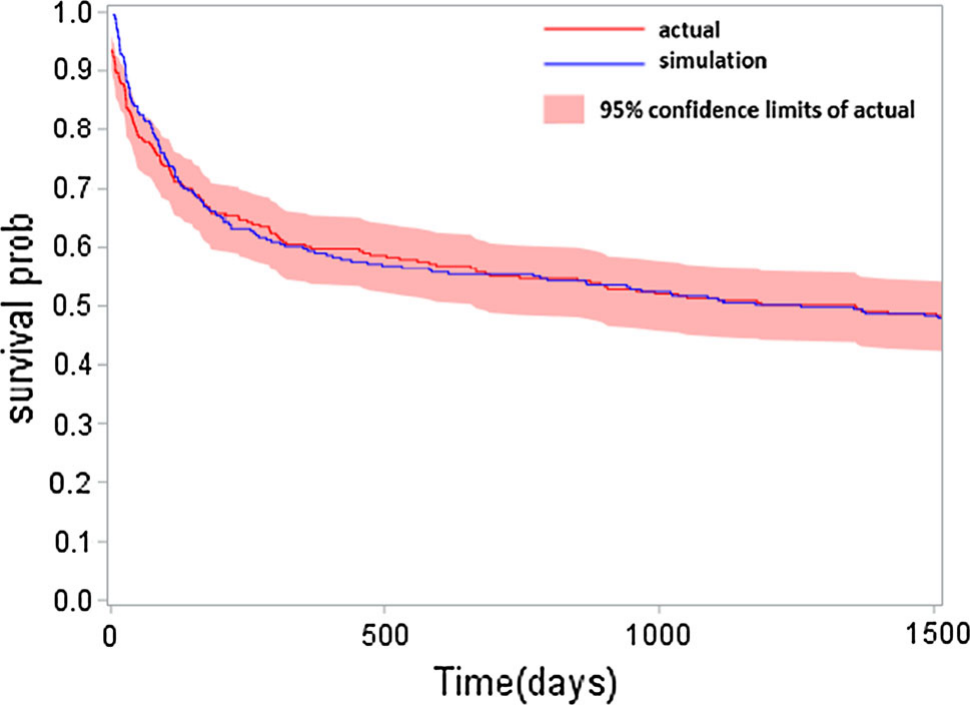
Survival curves of empirical and probabilistic models

With respect to face validity, the model structure, data source, and results were corroborated by consultant haematologists (CB and RP). For internal validity, the simulated/predicted outcomes were compared to empirical estimates derived from HMRN. The survival curves over a 5-year period are compared in Fig. 5. As shown, the simulated results closely match the 5-year follow-up empirical data, demonstrating good fit with empirical evidence. With regard to medical costs, the average 5-year simulated cost was £18,096 per patient (ranging from £18,078 to £18,114 among 500 iterations), capturing 98 % of the empirical results derived from the study population (£18,515).

Regarding external validity, the simulated cost and survival results were broadly similar to the findings in earlier literature [8, 10, 16, 17]. Taking the average costs of first-line treatment with R-CHOP as an example: for those over 60 years of age, our 5-year result of £20,831 is close to the US estimate of £19,485 [16] and the UK estimate of £19,805 [10], but less than the Canadian estimate of £26,498 [8]. For those under 60 years of age, our 15-year result of £26,761 is close to the Canadian estimate of £28,626 [8], but more than the UK estimate of £20,798 [10] (all currencies were inflated and converted to 2013 British pound sterling). Regarding the average survival time following R-CHOP, for those over 60 years of age, our 15-year result of 7.87 years is close to the estimate of 6.2 years from Johnston’s study [8] and the estimate of 6.23 years from Knight’s study [10]. For those under 60 years of age, our 15-year result of 11.8 years is slightly more than Knight’s estimate of 9.9 years [10] and Johnston’s estimate of 8.3 years [8]. Thus, overall, our model demonstrated good capability for predicting both medical costs and life expectancy. For more details, please refer to online Supplementary Table 7.

## Discussion

This is the first DLBCL model to simulate and predict treatment costs and life-years gained throughout the treatment pathway. Whilst several economic models have previously been constructed and published for DLBCL, all were built for the purpose of assessing the cost-effectiveness of adding rituximab to CHOP, not for overall disease treatments [8–10, 14–16]. Hence, although these reports confirmed the fact that adding rituximab to CHOP was cost-effective, none could examine the overall economic impact to health insurers or policy makers. In addition, this is the first DLBCL model to incorporate data on patients who were not treated with curative intent; enabling the production of more accurate estimates of the economic impact of DLBCL among different patient groups, as well as across the patient population as a whole. This flexibility allowed quantities such as the prevalence-based economic impact at a national level to be explored for the first time.

In the current study, a discrete event simulation (DES) model was built for analysis. The DES model generated individual treatment histories within the set time horizon for hypothetical DLBCL patients based on ‘real-world’ observational data. Estimates for the 5-year expected medical cost and life-years gained were £18,096 and 2.8 years, respectively, while the lifetime expected medical cost and survival were £18,396 and 10.0 years, respectively. Curative treatment results in a high number of life-years gained at the relatively moderate cost of £1535 per life-year gained (95 % CI £1534–£1537 per life-year gained) compared to non-curative care only. The variability was driven largely by initial treatment, age at diagnosis, and whether the patient had an ASCT. The expected lifetime medical cost ranged from £79,131 to £2930, while the life expectancy ranged from 30 days to 19 years (Table 3).

With respect to appropriate time horizons, this study demonstrated that the differences in estimated costs derived from 5-year, 15-year, and lifetime horizons were relatively minor, reflecting the high response rate amongst treated patients. However, the differences in estimated life-years gained were considerable (Table 3); confirming, as has been suggested by others [14], that the lifetime horizon is the optimal approach as it allows the overall effects of treatment to be fully captured. Furthermore, as expected, patients younger than 70 years had better survival and therefore incurred higher medical costs (Fig. 3). Importantly, however, in the 5-year time frame the differences in costs and life-years gained between these two age groups were small, demonstrating that patients over 70 years who receive chemotherapy responded as well as their younger counterparts. In this context, it is important to note that a patient’s performance status has been shown to be more discriminatory of survival than chronological age [25]. Finally, although the average cost of treating DLBCL is considered moderate in comparison to some other cancers (£18,396, see Table 1), the annual economic impact of treating existing and new DLBCL patients in the UK is considerable (in the region of £88–£92 million, see Fig. 4); accounting for approximately 1/6th of the annual UK NHS budget for haematological diseases as a whole [26] and providing around 35,000 life-years gained per year (data not shown).

Predicated on ‘real-world’ data, this model produced findings that can be extrapolated to the general patient population; which is not the case for models built using data from clinical trials [9–12, 14, 16]. The reliability and robustness of the model are also confirmed by the internal and external validations. For internal validation, the simulated cost and life-year results were close to the empirical HMRN data (Fig. 5). For external validation, the model results were in line with the findings from other observational studies: the micro-costing study conducted in Canada [17] and the Medicare claims study conducted in the US [16]. Moreover, the structure of our DES model provided an opportunity to make detailed comparisons with findings from more restricted datasets: and when the parameters (e.g. specific age groups, chemotherapy regimens and time horizons) from those studies were applied to the model, the results were found to be in line with most of the relevant studies [8, 10, 16, 17] (please refer to “Model validation”).

The molecular heterogeneity of DLBCL coupled with recent advances in diagnostic technologies [27, 28] is resulting in the development of more targeted approaches to the treatment of this complex cancer. The recent UK Phase 3 trial (REMoDL-B), for example, used gene expression profiling to assign DLBCL patients to different treatment arms [29]; and, as science continues to advance and new treatments emerge, reliable models such as the one developed here will become increasingly important in this rapidly changing field. Furthermore, the reduced side-effects and toxicities of many of these novel therapeutic agents mean that the proportion of the patient population who receive treatment will continue to increase. Accordingly, our model’s ability to accurately predict the impact of these changes across the entire population of patients with DLBCL will not only support commissioners to allocate resources, but will also aid clinical decision making.

With respect to model limitations, the current study did not deal with an administrative censoring effect. However, this is unlikely to impact on results, as the degree of administrative censoring is non-informative (independent of treatment). Also, the model is validated by the results which are broadly similar to the findings in earlier literature. In addition, whilst cost inputs were mainly confined to inpatient and day-case settings, the majority of costs will have been captured since around 85 % of cancer spending is incurred in acute/secondary care settings [26]. Moreover, as the model is based on an empirical follow-up time of only 5 years, the results for treated patients need to be interpreted with caution. Nonetheless, as the majority of treatments are carried out within the first 5 years, the costs estimates will be largely unaffected (see Table 3). It is, however, worth noting that our long-term estimates are conservative since they do not include health care costs for comorbidities that could potentially have been caused by DLBCL therapy. Quality adjusted life years (QALY) were not used in the model, as this information was not available for this cohort of patients and currently no other high quality QALY information is published in the existing literature, an issue recognised by other researchers [10, 15]. However, the model has been built to allow QALY data to be incorporated in the future and we are now collecting European Quality of Life-5 Dimensions (EQ-5D) information from patient cohort members.

## Conclusion

Life expectancy and costs of treating DLBCL patients vary according to patient characteristics and treatment pathways. However, the population-based model developed in the current study demonstrated a good capability of capturing the medical costs to healthcare commissioners, as well as the life-years gained in a real world setting. Importantly, the model produces different outputs for different purposes; estimating total costs along with health benefits at varying time points for specific patient cohorts, as well as generating prevalence-based costs for all patients over specific time periods. Future application of the model could include evaluation of new technologies/treatments to support healthcare decision makers, especially in the era of personalised medicine.

## Electronic supplementary material

Below is the link to the electronic supplementary material.

Supplementary material 1 (PDF 471 kb)
