# Supplementary material for: Treatment cost and life expectancy of diffuse large B-cell lymphoma (DLBCL): a discrete event simulation model on a UK population-based observational cohort
Source: Eur J Health Econ. 2016 Mar 11;18(2):255–67. doi: 10.1007/s10198-016-0775-4 (PMC5313581; doi:10.1007/s10198-016-0775-4)
Supplement: Supplementary file 1 — Supplementary material 1 (PDF 471 kb) [file 10198_2016_775_MOESM1_ESM.pdf]

The European Journal of Health Economics

Treatment Cost and Life Expectancy of Diffuse Large B-Cell Lymphoma (DLBCL)

A Discrete Event Simulation Model on a UK Population Based Observational Cohort

Han-I Wang, Alexandra Smith, Eline Aas, Eve Roman, Simon Crouch, Cathy Burton, Russell Patmore

Corresponding Author: Han-I Wang

Epidemiology & Cancer Statistics Group (ECSG), Department of Health Sciences, Seebohm Rowntree Building, University of York, Heslington, York, YO10 5DD, UK

Tel: +44 (0)1904 321896

Han-i.wang@york.ac.uk

Supplementary Tables

This Supplementary Appendix contains the information referred to the full version of this article.

Supplementary Table 1: patient characteristics

	Overall		Treated		Not treated	
	N	%	N	%	N	%
Total	271	100	217	100	54	100
Age at diagnosis (years)						
Median (Q1-Q3)	70	(59-79)	67	(57-76)	80	(75-86)
Sex						
Male	153	56.5	127	58.5	26	48.2
Female	118	43.5	90	41.5	28	51.8
International Prognostic Index (IPI)						
Low (0-1)	55	20.3	55	25.4	-	-
Low-Intermediate (2)	43	15.9	43	19.8	-	-
Intermediate-High (3)	47	17.3	43	19.8	4	7.4
High (4-5)	78	28.8	54	24.9	24	44.4
Not known	48	17.7	22	10.1	26	48.2
Age (years) >60						
Yes	194	71.6	144	66.4	50	92.6
No	77	28.4	73	33.6	4	7.4
Stage						
I	41	15.1	41	18.9	-	-
II	39	14.4	37	17.1	2	3.7
III	33	12.2	32	14.7	1	1.8
IV	132	48.7	102	47.0	30	55.6
Not fully staged	26	9.6	5	2.3	21	38.9
Extranodal Site >1						
Yes	89	32.8	69	31.8	20	37.0
No	156	57.6	142	65.4	14	26.0
CT scan not done	26	9.6	6	2.8	20	37.0
Performance status (ECOG)						
0	48	17.7	47	21.7	1	1.8
1	129	47.6	114	52.5	15	27.8
>1	94	34.7	56	21.8	38	70.4
Elevated Lactose Dehydrogenase (LDH)						
Yes	158	58.3	129	54.4	29	53.7
No	69	25.5	65	30.0	4	7.4
Not measured	44	16.2	23	10.6	21	38.9
Status at 5 years after diagnosis						
Alive	125	46.13	125	57.6	-	-
Dead	146	53.87	92	42.4	54	100
First line treatment						
Standard	161	59.41	162	74.7	-	-
Stage IA	25	9.23	25	11.5	-	-
CNS prophylaxis	23	8.49	23	10.6	-	-
CNS involvement	7	2.58	7	3.2	-	-
Not treated	54	19.9	-	-	54	100

Supplementary Table 2: Parameters for first line treatment

Regimen	CHOP	R-CHOP	R-CVP	CODOX-M/IVAC-R	IDARAM	Radiotherapy only
Treatment type						
Age ≤ 60						
% stage IA	11	89	0	0	0	0
% CNS involvement	0	20	0	20	60	0
% standard	4	96	0	0	0	0
Age 60-80						
% stage IA	8	77	0	0	0	15
% CNS involvement	0	100	0	0	0	0
% standard	5	86	9	0	0	0
Age ≥ 80						
% stage IA	0	100	0	0	0	0
% CNS involvement	0	100	0	0	0	0
% standard	4	56	28	0	0	0
Standard number of cycles	3 or 6	6 or 8	8	2	2	20 fractions
% Achieved full cycles						
Age ≤ 60	67	85	0	100	100	100
Age 60-80	20	76	44	100	100	80
Age ≥ 80	0	50	28	0	0	50
(1st cycle) % treated as inpatient	60	39	56	100	100	0
(1st cycle) Average (SD) LOS	22 (12)	11 (14)	9 (7)	31 (34)	37 (15)	-
(1st cycle) Min-Max of LOS	8-48	1-68	1-20	7-55	19-68	-
(Subsequent cycles) % treated as inpatient	0	4	0	100	40	0
(Subsequent cycles) Average (SD) LOS	19 (10)	9 (12)	10 (4)	7 (2)	30 (12)	-
(Subsequent cycles) Min-Max of LOS	2-25	1-62	7-13	6-8	7-54	-
Drug cost (per cycle/per fraction)	£289	£1,730	£1,486	£6,241	£2,006	£123

* CHOP: cyclophosphamide, doxorubicin, vincristine and prednisolone
R-CHOP: cyclophosphamide, doxorubicin, vincristine and prednisolone + rituximab
R-CVP: cyclophosphamide, vincristine, prednisolone + rituximab
CODOX-M/IVAC-R: rituximab, cyclophosphamide, vincristine, doxorubicin, and methotrexate + rituximab, etoposide, ifosfamide and cytarabine
IDARAM: idarubicin, dexamethasone, cytarabine, methotrexate
CNS: central nervous system
LOS: length of stay

Supplementary Table 3: Parameters for second line treatment

Regimen	R-CHOP	R-DHAP	R-ESHAP	R-CVP	R-GCVP	CODOX-M/IVAC-R	IDARAM	Radiotherapy only
Standard number of cycles	3	3	3	6	6	2	2	20 fractions
% Achieved full cycles								
Age ≤ 60	100	46	100	0	0	0	67	100
Age 60-80	0	14	100	0	0	0	00	80
Age ≥ 80	0	0	0	0	0	0	0	50
(1st cycle) % treated as inpatient	33	39	100	0	0	100	100	0
(1st cycle) Average (SD) LOS	4 (2)	11 (14)	17 (15)	11 (4)	12 (4)	31 (34)	37 (15)	-
(1st cycle) Min-Max of LOS	2-6	1-68	6-28	2-15	2-14	7-55	19-68	-
(Subsequent cycles) % treated as inpatient	25	4	100	0[†]	0[†]	100	40	0
(Subsequent cycles) Average (SD) LOS	20 (10)	9 (12)	14 (9)	11 (4) [†]	12 (4) [†]	7 (2)	30 (12)	-
(Subsequent cycles) Min-Max of LOS	2-25	1-62	7-20	2-15[†]	2-14[†]	6-8	7-54	-
Drug cost (per cycle/per fraction)	£1,730	£1,952	£3,344	£1,486	£1,593	£6,241	£2,006	£123

* R-CHOP: cyclophosphamide, doxorubicin, vincristine and prednisolone + rituximab
R-DHAP: dexamethasone, cytarabine, cisplatin + rituximab
R-ESHAP: etoposide, methylprednisolone, cytarabine, cisplatin + rituximab
R-CVP: cyclophosphamide, vincristine, prednisolone + rituximab
R-GCVP: gemcitabine, cyclophosphamide, vincristine, prednisolone + rituximab
CODOX-M/IVAC-R: rituximab, cyclophosphamide, vincristine, doxorubicin, and methotrexate + rituximab, etoposide, ifosfamide and cytarabine
IDARAM: idarubicin, dexamethasone, cytarabine, methotrexate
LOS: length of stay

[†] Assume the results are similar to the 1st cycle due to lack of data

Supplementary Table 4: Parameters for third line treatment

Regimen	MiniBEAM	Methotrexate
Standard number of cycles	2	2
% Achieved full cycles		
Age ≤ 60	100	80
Age 60-80	100	80
Age ≥ 80	100	50
(1st cycle) % treated as inpatient	70	100
(1st cycle) Average (SD) LOS	20 (8)	4 (1)
(1st cycle) Min-Max of LOS	9-26	4-4
(Subsequent cycles) % treated as inpatient	67	100
(Subsequent cycles) Average (SD) LOS	21 (4)	4 (1)
(Subsequent cycles) Min-Max of LOS	9-30	4-4
Drug cost (per cycle)	£446	£861

* MiniBEAM: carmustine, etoposide, cytarabine, melphalan
LOS: length of stay

Supplementary Table 5: cost items

Phases	Cost items
**Diagnosis**	Scans
	Biopsies
	ECG (Electrocardiography)
	ECHO (Echocardiograph)
	Blood tests
**First line treatment**	Chemotherapy:
	CHOP
	CHOP/R
	CVP/R
	CODOX-M/IVAC-R
	IDARAM
	Radiotherapy only
	Additional treatment:
	Methotrexate
	Radiotherapy
	Supportive care:
	G-CSF
	Transfusion
	Post chemotherapy imaging
**Second line treatment**	Chemotherapy:
	CHOP/R
	DHAP/R
	ESHAP/R
	GCVP/R
	CVP/R
	CODOX-M/IVAC-R
	IDARAM
	Radiotherapy only
	Additional treatment:
	Methotrexate
	Radiotherapy
	Supportive care:
	G-CSF
	Transfusion
	Post chemotherapy imaging
**Third line treatment**	Chemotherapy:
	Mini-BEAM
	Methotrexate
	Supportive care:
	G-CSF
	Transfusion
	Post chemotherapy imaging
**Follow up**	Out-patient visits
	Imaging if patients relapsed
	Biopsies if patients relapsed
**End-of-life care**	In-patient treatment
	Out-patient visits

CHOP (cyclophosphamide, doxorubicin, vincristine and prednisone); R-CHOP (cyclophosphamide, doxorubicin, vincristine, prednisone and rituximab); R-CVP (cyclophosphamide, vincristine, prednisone and rituximab); CODOX-M/IVAC-R (cyclophosphamide, doxorubicin, vincristine, methotrexate/ifosfamide, etoposide, high dose cytarabine and rituximab); IDARAM (rituximab, idarubicin, dexamethasone, cytarabine and methotrexate); R-DHAP (dexamethasone, cytarabine, cisplatin and rituximab); R-ESHAP (etoposide, methylprednisolone, cytarabine, cisplatin and rituximab); MiniBEAM (carmustine, etoposide, cytarabine and melphalan).

Supplementary Table 6: details of survival models

	Response after 1st line treatment		Response after 2nd line treatment		End of life care	
	**Time to relapse**	**Time to death**	**Time to relapse**	**Time to death**	**Treated**	**No treated**
**Model type**	Weibull	Exponential	Exponential	Exponential	lognormal	Lognormal
**Intercept**	10.73	12.31	32.36	8.49	5.76	-0.20
**Age**	0.09	-0.05	-0.1	-	-0.03	0.04
**Time in treatment**	-0.009	-	-	0.01	-	-
**Had autologous stem cell transplant or not**	-	-	-18.44	-	-	-
**Achieved full cycle of treatment or not**	-	-	-2.5	-	-	-
**Shape parameter**	0.60	-	-	-	-0.43	0.20

Supplementary Table 7: Comparison table of simulated and relevant study results

	Country	Price year	Patient age	Model approach	Discount rate	Time horizon	Regimen	Mean Cost (2013 UK pound *)	Mean time	Simulated cost	Simulated time
Hornberger et al.	USA	2003	>=60	Markov	3%	5 year	CHOP	$7308 (£6394)	3.11 yrs	£7,163	1.78 yrs
							CHOP/R	$22430 (£19485)	4.15 yrs	£20,831	3.52 yrs
Johnston et al.	Canada	2006	>15	Discrete event simulation	3%	15 year	<60				
							CHOP	$36765 (£22713)	7.7 yrs	£14525	8.3 yrs
							CHOP/R	$46337 (£28626)	8.3 yrs	£26761	11.8 yrs
							>60				
							CHOP	$34968 (£21603)	4.5 yrs	£7163	1.78 yrs
							CHOP/R	$42892 (£26498)	6.2 yrs	£20831	7.87 yrs
Knight et al.	UK	2004	Unclear	Markov	6% cost<br>1.5% outcome	15 year	<60				
							CHOP	£7311 (£10016)	8.86 yrs	£14525	8.3 yrs
							CHOP/R	£15181 (£20798)	9.90 yrs	£26761	11.8 yrs
							>60				
							CHOP	£5773 (£7909)	5.25 yrs	£7163	1.78 yrs
							CHOP/R	£14456 (£19805)	6.23 yrs	£20831	7.87 yrs
Best et al.	France	2003	60-80	Markov	3%	15 year	CHOP	€28782 (£28079)	4.84 yrs	£8364	2.2 yrs
							CHOP/R	€41952 (£40927)	5.83 yrs	£21596	8.58 yrs
Groot et al.	Netherlands	2002	unclear	Markov	4%	15 year	<60				
							CHOP	€27828 (£25360)	6.91 yrs	£14525	8.3 yrs
							CHOP/R	€40171 (£36609)	7.74 yrs	£26761	11.8 yrs
							>60				
							CHOP	€26891 (£24506)	4.17 yrs	£7163	1.78 yrs
							CHOP/R	€42751 (£38960)	5.14 yrs	£20831	7.87 yrs
Lee et al.	Canada	2004	21-90	Micro-costing	0%	-	CHOP:		-		-
							1st line	$12240 (£7043)		£4625	
							Total	$35292 (£20307)		£10461	
							CHOP/R				
							1st line	$33088 (£13897)		£15862	
							Total	$47377 (£27261)		£23043	

* All currencies were inflated and converted to 2013 UK sterling.
